# Assessment of the Potential for Genomic Selection To Improve Husk Traits in Maize

**DOI:** 10.1534/g3.120.401600

**Published:** 2020-08-14

**Authors:** Zhenhai Cui, Haixiao Dong, Ao Zhang, Yanye Ruan, Yan He, Zhiwu Zhang

**Affiliations:** * College of Biological Science and Technology, Liaoning Province Research Center of Plant Genetic Engineering Technology, Shenyang Key Laboratory of Maize Genomic Selection Breeding, Shenyang Agricultural University, Shenyang 110866, China; † Dept. of Crop and Soil Sciences, Washington State University, Pullman, WA 99164; ‡ College of Plant Sciences, Jilin University, Changchun 130062, China; § National Maize Improvement Center of China, Beijing Key Laboratory of Crop Genetic Improvement, China Agricultural University, Beijing, 100094, China

**Keywords:** genomic selection, husk, population structure, prediction accuracy, maize, gBLUP, marker assisted selection, breeding, rrBLUP, GAPIT, GenPred, Genomic, Prediction, Shared data resources

## Abstract

Husk has multiple functions such as protecting ears from diseases, infection, and dehydration during development. Additionally, husks comprised of fewer, shorter, thinner, and narrower layers allow faster moisture evaporation of kernels prior to harvest. Intensive studies have been conducted to identify appropriate husk architecture by understanding the genetic basis of related traits, including husk length, husk layer number, husk thickness, and husk width. However, marker-assisted selection is inefficient because the identified quantitative trait loci and associated genetic loci could only explain a small proportion of total phenotypic variation. Genomic selection (GS) has been used successfully on many species including maize on other traits. Thus, the potential of using GS for husk traits to directly identify superior inbred lines, without knowing the specific underlying genetic loci, is well worth exploring. In this study, we compared four GS models on a maize association population with 498 inbred lines belonging to four subpopulations, including 27 lines in stiff stalk, 67 lines in non-stiff stalk, 193 lines in tropical-subtropical, and 211 lines in mixture subpopulations. Genomic Best Linear Unbiased Prediction with principal components as cofactor, performed the best and was selected to examine the impact of interaction between sampling proportions and subpopulations. We found that predictions on inbred lines in a subpopulation were benefited from excluding individuals from other subpopulations for training if the training population within the subpopulation was large enough. Husk thickness exhibited the highest prediction accuracy among all husk traits. These results gave strategic insight to improve husk architecture.

The husk is a leaf-like tissue covering the outside of a maize ear. Similar to rice and coconut husks (Ali 2011; Ding *et al.* 2012; Johar *et al.* 2012), maize husks recycle anthocyanins and provide fiber for secondary uses such as bioethanol production(Li *et al.* 2008; Jalil *et al.* 2012; Ekhuemelo and Tor 2013). More specifically, the maize husk performs three major physiological functions. First, the husk performs limited photosynthesis to provide carbon with a C_4_-like pathway(Pengelly *et al.* 2011; Wang *et al.* 2013). Second, the husk protects the ear from pest damage and pathogen infection(Barry *et al.* 1986; Warfield and Davis 1996; Demissie *et al.* 2008). Particularly, in subtropical or tropical areas, ear rot is a serious issue during maize ear development (Renfro and Ullstrup 1976; Afolabi *et al.* 2007). Tight-husked maize is more resistant to ear rot than loose-husked maize(Warfield and Davis 1996). Third, the husk prevents moisture from penetrating the maize ear before harvest time(Sweeney *et al.* 1994). After physiological maturation of maize kernels, the husk is the main pathway for kernel dehydration. In temperate areas, the timing of mechanical harvest requires fast dehydration of maize during colder weather(Hicks *et al.* 1976). Thus, appropriate husk architecture is essential to both ear development and kernel dehydration prior to harvest.

Although much morphological and genetic research of the maize husk has been conducted during the past decades, molecular breeding studies of husk traits are still in their infancy. Husk development initiates from the lateral meristem(Wang *et al.* 2013). The main traits that influence the level of husk function are husk length (HL), layer number (HN), thickness (HT) and width (HW). HN typically ranges from 6 to 19 in hybrids and inbreds(Brewbaker and Kim 1979). HN is highly correlated to tassel branch number(Brewbaker 2015). The variance in husk traits is large across both natural populations and recombinant inbred lines (RIL) (Brewbaker and Kim 1979; Zhou *et al.* 2016a; Cui *et al.* 2016, 2018). The husk traits are genetically controlled by multiple genes. In our recent study(Cui *et al.* 2018), we dissected the genetic architecture of HL, HN, and HW across three RIL populations. We detected a total of 21 quantitative trait loci (QTL) associated with the three husk traits. In most cases, the associations included one or two large-effect QTL plus many small-effect QTL. In another previous study in a maize association inbred population, we detected 63 single nucleotide polymorphisms (SNPs) by genome-wide association study(GWAS) that were significantly associated with HL, HN, HT, and HW (*P* < 1.04×10^−5^)(Cui *et al.* 2016). However, none of these SNPs passed the classic, standard threshold of α = 0.01 after Bonferroni correction(Holm 1979). Similarly, another GWAS of HN and husk weight did not find any associated loci under the same threshold(Zhou *et al.* 2016a). Based on the evidence above, husk traits are complex and governed by many genes, most with small effects which are hard to detect through GWAS. Consequently, molecular breeding using traditional marker-assisted selection (MAS) is inefficient for these polygenic, complex traits because MAS works best with QTL that have large or moderate effects(Collard and Mackill 2008; Hayes *et al.* 2009).

Genomic selection (GS) was developed in 1990s (Bernardo 1994) based on mixed linear model and expanded to Bayesian framework in 2000s (Meuwissen *et al.* 2001). Genotyping cost reduction due to newly developed molecular technologies made GS more affordable for breeding(Zhang *et al.* 2007; VanRaden 2008). GS is efficient in improving polygenic traits controlled by small-effect genetic loci, such as animal body size(VanRaden *et al.* 2009; Chesnais *et al.* 2016; Mehrban *et al.* 2017) and plant yield(Crossa *et al.* 2013, 2014). GS incorporates all marker effects across the whole genome to evaluate genomic estimated breeding values (GEBVs). With a training set that includes both genotypic and phenotypic data, a prediction model is “trained” to calculate GEBVs for the validation set (also called the testing set). GS was introduced in 1994 in format of the genomic Best Linear Unbiased Prediction (gBLUP) and maize was used for the demonstration. One study in a bi-parental maize population found that estimates of stover and grain yield using GS were 14–50% higher than with MAS(Massman *et al.* 2013). Another study in a multi-parental maize population found that GS achieved genetic gains of ∼2% for grain yield with two rapid cycles per year(Zhang *et al.* 2017a).

Prediction accuracy, defined as the correlation between the observed and predicted breeding values, is commonly used to assess the efficiency of GS(Combs and Bernardo 2013).Prediction accuracy in plant breeding is dependent on statistical models used(Heffner *et al.* 2009; Ogutu *et al.* 2011; Spindel *et al.* 2015), training population size(Heffner *et al.* 2011), the relationship between the training and testing populations(Ly *et al.* 2013), marker density(Zhang *et al.* 2017b), rate of linkage disequilibrium decay(Calus and Veerkamp 2007), and trait heritability(Dong *et al.* 2018). Trait heritability can be defined as broad-sense or narrow-sense. Broad-sense heritability includes all genetic contributions, including additive, dominant, and epistatic effects. Narrow-sense heritability includes only additive effects, which is more important for breeding with selection based on additive effects(Holland *et al.* 2010).

Population structure is also considered an essential factor influencing prediction accuracy(Ly *et al.* 2013; Guo *et al.* 2014). Several strategies have been compared to handle population structure in GS. One cassava study evaluated prediction accuracy by comparing cross-validation with close relatives (CV-CR) to cross-validation without close relatives (Ly *et al.* 2013). In a similar comparison on a maize association panel, three strategies were evaluated: within subpopulations, across subpopulations, and combined subpopulations(Guo *et al.* 2014). The closer genetic relationship among individuals of CV-CR and individuals within subpopulations have closer genetic relationships led to higher prediction accuracy.

Maize has a strong population structure, typically classified as Stiff Stalk (SS), Non-Stiff Stalk (NSS), Tropical-SubTropical (TST), and MIXED (MIXED) subpopulations. Our objectives are 1) to demonstrate that MAS is less favorite for prediction on husk traits, 2) to model population structure appropriately to increase prediction accuracy, and 3) to investigate the interactions between training population size and subpopulations. The results are expected to provide the inside to improve husk traits through GS, especially to make a strategic plan for selecting traits and establishing training populations across subpopulations.

## Materials And Methods

### Plant materials and husk trait observations

The maize association panel used in this study is comprised of 508 inbred lines, which were collected from tropical, subtropical, and temperate germplasms(Yang *et al.* 2011; Li *et al.* 2012). Due to germination issues, only 498 lines were measured in this experiment. These inbred lines were categorized into four subpopulations based on origins: 27 SS lines, 67 NSS lines, 193 TST lines, and remaining 211 MIXED lines. Detailed information about this panel is listed in supplemental material (Table S4).

All the lines were planted as single row plots with three replications using a randomized complete block design in two locations in China: Sanya (SY) city of Hainan (HN) province in southern China in 2013 and Beijing (BJ) city in northern China in 2014. All plants were grown under open-pollination conditions. Four traits, HN, HL, HT, and HW, were measured at the maturity stage from at least six well-pollinated plants in each row in both locations. HN was counted from the first (outer) layer of each husk to the last layer (inner). HL was measured on the third layer of each husk (counting from the outside to inside). HW was measured at the midpoint of the third layer of each husk. HT was measured as total thickness by punching a disc from the interior to the exterior of all husk layers. Husk phenotype data are listed in supplemental material (Table S4). Detailed husk measurement information is provided in the previous study (Cui *et al.* 2016). Mean trait values of all replications were calculated across all environments. We used mean values instead of BLUPs for two reasons. One is that mean values and BLUPs are similar in balanced data. The other is to avoid contamination of using information from testing population. (Dong *et al.* 2018).

### Genotypic markers

Genotypic markers for the 508 lines were downloaded from the website of www.maizego.org/Resources, with the download link: https://pan.baidu.com/s/1mhR1L1Y#list/path=%2F. The genetic markers came from four genotyping platforms: the Illumina Maize SNP50 array, RNA sequencing, reduced genome sequencing (GBS), and the Affymetrix Axiom Maize 600K array. It took three steps to merge all SNP markers for each platform. First, Yang *et al.* (2014)(Yang *et al.* 2014) combined the 56,110 SNPs from the SNP array and the 556,809 SNPs from RNA sequencing(Fu *et al.* 2013), using identity by descent based projection and the k-nearest neighbor algorithm. Second, Liu *et al.* (2017)(Liu *et al.* 2017) conducted SNP allele calling by GBS and the 600K SNP array. The missing genotypes were imputed by Beagle v4.0(Browning and Browning 2007). Third, quality control was applied to remove SNPs with minor allele frequencies below 5%. The final dataset was composed of ∼1.25 M SNPs.

### Estimates of heritability, genomic breeding values, and genomic selection

The four husk traits were analyzed one at a time for each environment and their mean values across environments separately in a fixed and random effects mixed linear model. The first three principal components (PCs) derived from all markers were fitted as fixed effects. The additive genetic effects of individuals and the residuals were fitted as random effects. The statistical model is as follows:

y =μ+ Xβ+ Zu +ε (1)

where y is a vector (n*1) of observations, and n is the number of lines; μ is the overall mean; β is a vector (p*1) of fixed effects; **u** is a vector (n*1) of random effects representing additive genetic effects of individuals; **X** is a design matrix (n*p) for fixed effects, so p equals 3 when the first three PCs were used as cofactors; **Z** is a design matrix (n*n) for random line effects, so **Z** is the identity matrix; and ε is the residuals. The random effects followed normal distributions: **u** ∼ *N*(0, Kσ_u_^2^) and ε ∼ *N*(0, Iσ_e_^2^), where I is the identity matrix and **K** is the additive relationship matrix (n*n) derived from all the markers using Zhang algorithm in GAPIT(Lipka *et al.* 2012). σ_u_^2^ is the variance of individual additive genetic effects, and σ_e_^2^ is the variance of residuals. The estimation of **u** is the estimated genomic breeding values. The proportion of σ_u_^2^ over the total variance (σ_u_^2^ + σ_e_^2^) was defined as the estimate of heritability. The analyses was conducted by using the R software packages, GAPIT(Lipka *et al.* 2012; Tang *et al.* 2016). The phenotypes and genotypes of all individuals were used in the analyses to estimate heritabilities and genomic breeding values. The model was also used as genomic selection with the whole population divided into training and testing populations.

### Model selection

Four models were evaluated for accuracy of prediction. The evaluations were conducted by randomly sampling 20% of whole population as testing population and the rest as training population. The first model is a MAS. GWAS were conducted in the training population using BLINK, a software implemented in C language (Huang *et al.* 2018). The top ten most significant associated SNPs, named as quantitative trait nucleotide (QTNs) were used to predict the breeding values for the individuals in the testing population(Guo *et al.* 2011); The second model is a fixed effect model containing the first three PCs derived from all SNPs. The effects of the PCs were estimated in the training population. The estimated effects were used to predict the breeding values of individuals in the testing population. The third model is a random effect mixed model containing the additive genetic effect of the individuals with variance structure defined by the kinship matrix derived from all SNPs. All individuals in both training and testing populations were included in the analyses. However, the phenotypes of the individuals from the testing population were masked by setting them as “NA”. Breeding values were estimated for all individuals. This model is commonly referred as genomic Best Linear Unbiased Prediction (gBLUP). The fourth model is a combination of the second and third in the format of fixed and random effects mixed linear model. The analyses of gBLUP were conducted using R package “rrBLUP v4.5”(Endelman 2011). Pearson correlation coefficient was calculated between observed and predicted phenotypes. The random sampling was replicated 20 times.

### Assessment of gBLUP accuracy

The objective of the assessment was to evaluate the impact of training population size and relationship between training and testing populations on prediction accuracy using gBLUP model. Cross validations were performed under three scenarios: 1) Subpopulations ignored, where 10–90% of individuals from the whole population were randomly selected as the testing population, with the remaining individuals as the training population; 2) prediction within subpopulations, where 10–90% of one subpopulation were randomly selected treated as the testing set, with the remaining individuals of this same subpopulation as the training population; and 3) prediction across subpopulations, where 10–90% of individuals from one subpopulation were randomly selected as the testing population, with the remaining individuals of this subpopulation and the other subpopulations as the training population. Prediction accuracy was calculated as the Pearson correlation coefficient between predicted values and true values of the testing population. Sampling were repeated 100 times for each scenario.

### Data availability

All phenotypic data and results data are included within the manuscript in supplementary. The genotypes (∼1.25M) used in this study are all publicly available at the website of Jianbing Yan (http://www.maizego.org/Resources.html), or through the direct download link (https://pan.baidu.com/s/1mhR1L1Y#list/path=%2F). Supplemental material available at figshare: https://doi.org/10.25387/g3.11829177.

## Results

### Husk trait heritability and phenotypic correlation

**Figure 1:**
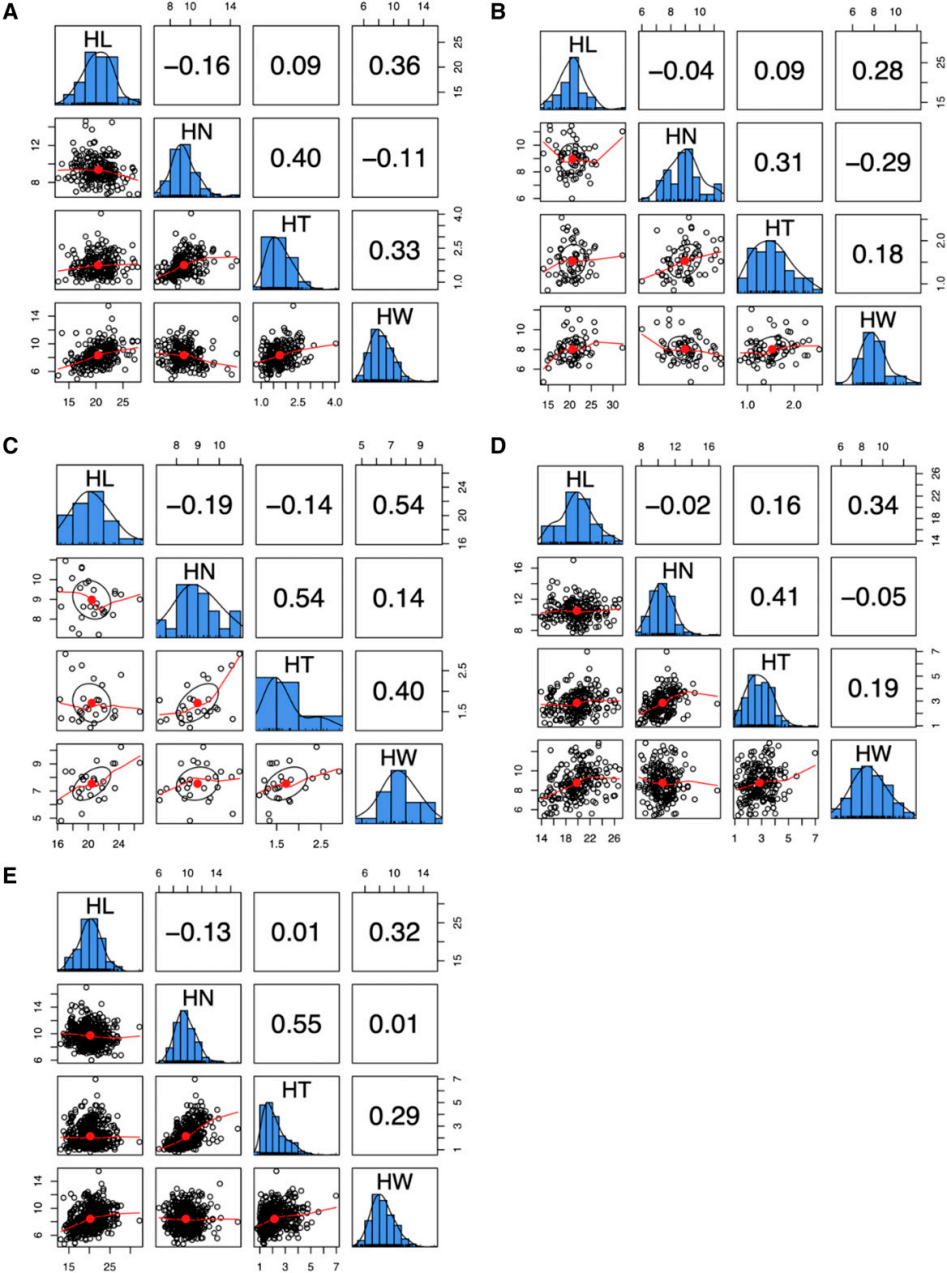
Phenotypic correlation and frequency distributions of four husk traits in different subpopulations. (A) Admixed (MIXED) subpopulation. (B) Non-stiff stalk (NSS) subpopulation. (C) Stiff stalk (SS) subpopulation. (D) Tropical-subtropical (TST) subpopulation. (E) Whole association panel. The husk traits are husk length (HL), husk layer number (HN), husk thickness (HT), and husk width (HW). The unit of measure is cm for HL, HT, and HW.The plots on the diagonal line exhibit the phenotypic distribution of the mean value for each husk trait. Displayed below the diagonal line, are the scatter plots for mean value of each two husk traits; displayed above the diagonal line are Pearson correlation coefficients. The red line and red dot represent the lowest regression fitting curve and the correlation ellipse, respectively.

All husk traits in the whole association populations or each subpopulation showed continuous and approximately normal distributions (Figure 1). In each subpopulation, HN and HT showed the highest correlation and the second were HL and HW. Within subpopulation, the highest correlation (r = 0.54) appeared in SS between HN and HT. The Estimated genomic breeding values followed the same trend (Figure S1).

**Figure 2:**
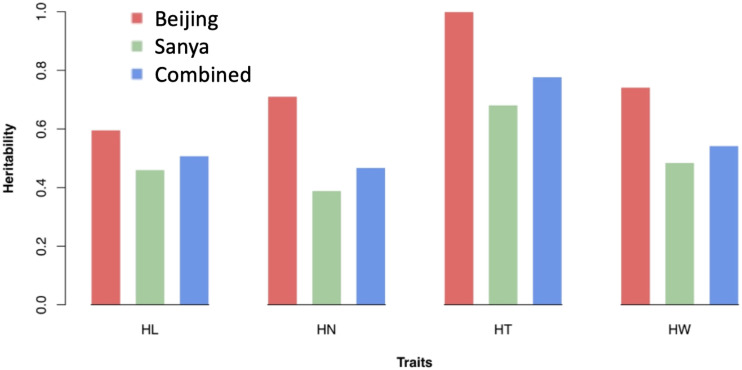
Heritability estimates of husk traits in two locations and combined. HL = husk length, HN = husk layer number, HT = husk thickness, and HW = husk width.

The narrow-sense heritability of husk traits was evaluated in two different locations separately and combined. In addition to residual effects, random effects are the individual total additive genetic effects with variance structure defined by an additive relationship matrix. All the husk traits showed higher heritability in Beijing than Sanya (Figure 2). The heritability of husk traits in Beijing ranged from 0.60 (HL) to 0.99 (HT). The heritability of husk traits in Sanya ranged from 0.39 (HN) to 0.68 (HT). The heritability of the combined locations ranged from 0.47 (HN) to 0.78 (HT). At each location, HT exhibited the highest heritability, ranging from 0.68 to 0.99.

### Model selection

**Figure 3:**
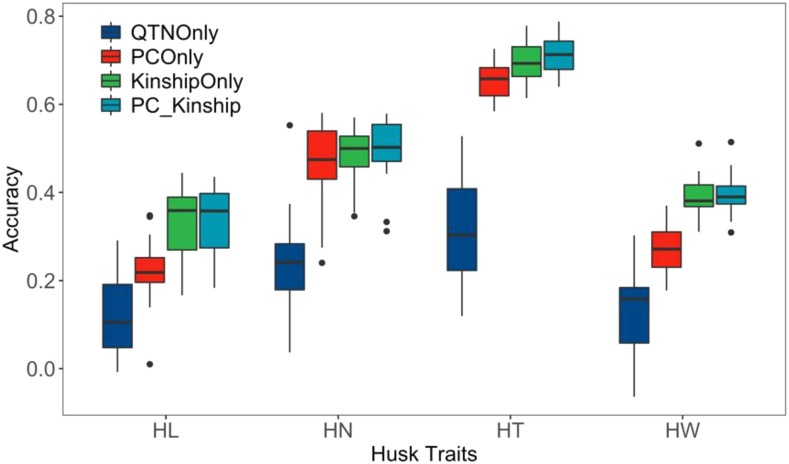
Comparison among four models to predict four husk traits. The comparisons were conducted in a population with 498 maize inbred lines by randomly sampling 20% of whole population as testing population and the rest as training population. The sampling was conducted 20 times. The models include 1) MAS using the top ten associated markers (QTNs) only; 2) Using PCs only; 3) Using kinship only; and 4) Using both PCs and kinship.

The association panel of 498 inbred lines has a strong population structure with four subpopulations. It was reasonable to have concerns if major associated SNPs or principal components would be close to sophisticated models such as gBLUP. Four models were compared: 1) MAS with the top ten associated SNPs; 2) PCs only; 3) Kinship only, and 4) PCs + Kinship. The results suggested MAS was far less accurate. PCs only were not as good as models with kinship. Kinship with PCs performed better than kinship across traits. The exception was HL, where the two models were similar (Figure 3). The results suggested that incorporating population structure only was not enough for prediction.

### Cross-validation by random sampling across whole population

We randomly masked 10–90% of all lines as the testing population (inference) and treated the remaining lines as the training population (reference, or validation). Prediction accuracy was computed as the Pearson correlation coefficient between the predicted and the observed phenotypes in the testing population using the instant method(Zhou *et al.* 2016b).

**Figure 4:**
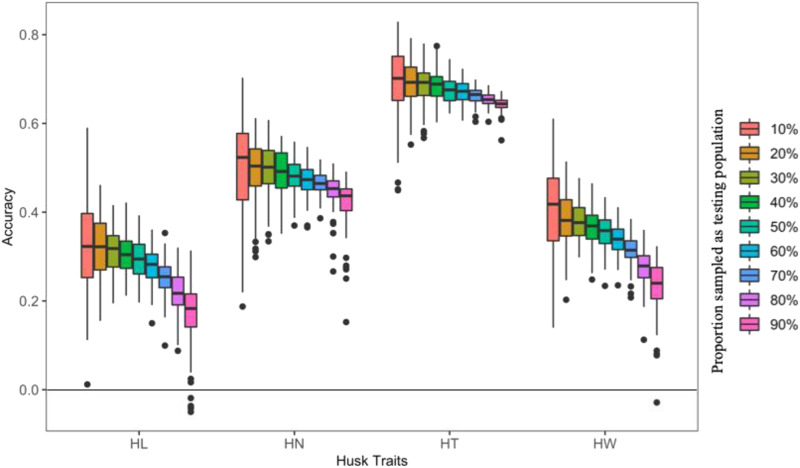
Accuracies to predict proportion of inbreds using the rest as training population. There are 498 maize inbred lines in total. Part of the lines (10–90%) were randomly sampled as the testing population and the rest as the training population. The sampling was conducted 100 times. HL = husk length, HN = husk layer number, HT = husk thickness, and HW = husk width.

HT and HL exhibited the highest and lowest prediction accuracy, respectively (Figure 4 and Table S1). In order from highest to lowest, the prediction accuracies for the four traits were HT > HN > HW > HL. In general, prediction accuracy declined as the proportion of inference increased for each husk trait. For HT, however, this decline was minimal, only decrease 7.3% from the highest and to the lowest. This may be because its heritability was higher in each environment compared to the other traits.

### Cross validation within subpopulations

**Figure 5:**
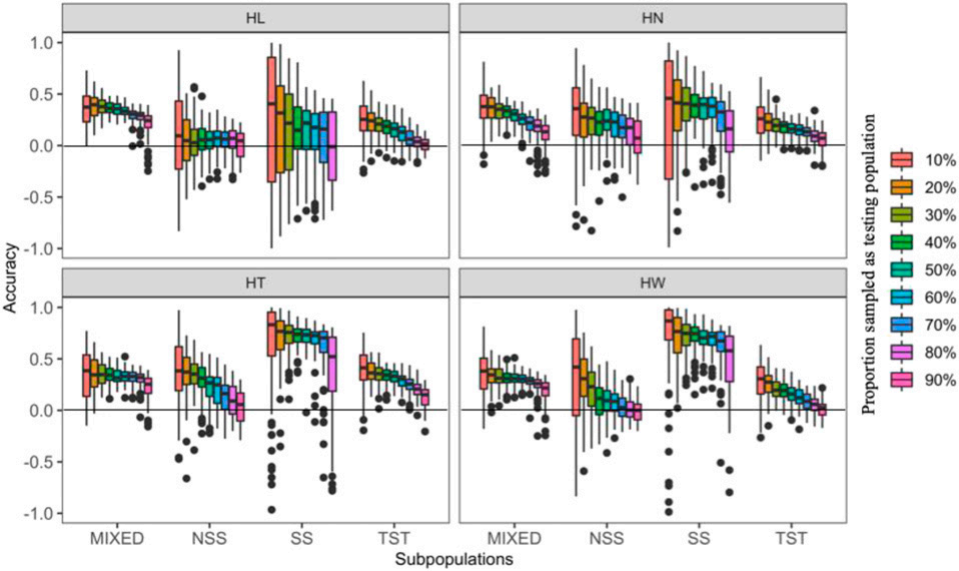
Prediction accuracies for four husk traits within subpopulations. There are 67 inbred lines in non-stiff stalk (NSS), 27 in stiff stalk (SS), 193 in ropical-subtropical (TST) and 211 in admixed (MIXED). Within each subpopulation, certain proportions (10–90%) of inbred lines were sampled as testing population and the rest lines within the subpopulation as the training population. The sampling was replicated 100 times. Husk traits: HL = husk length, HN = husk layer number, HT = husk thickness, and HW = husk width.

The degree of prediction accuracy in GS is related to the relationship between the training sets and the testing sets (Guo *et al.*, 2014; Ly *et al.*, 2013). GS within subpopulations that have closely related individuals will result in higher prediction accuracies than GS within randomly selected lines. Thus, to further estimate and compare prediction accuracies, we masked varying proportions (10–90%) of lines from a particular subpopulation into the testing population and treated the remaining lines within the same subpopulation as the training population (Figure 5 and Table S2). For example, the 10% proportion of inference within MIXED subpopulation represents sampling 10% lines from this subpopulation as testing population and remaining 90% lines from this subpopulation were treated as training population.

In the MIXED subpopulation, we found little difference in prediction accuracy among the husk traits using 10–50% of the inference population. In order from highest to lowest, the prediction accuracies for the four traits were HL > HN > HW > HT. In the NSS subpopulation, the order of prediction accuracies was HT > HW > HN > HL. In SS, HT and HW exhibited the higher levels of prediction accuracies compared to HN and HL, across all proportions used for the inference population. In TST, the highest prediction accuracies occurred with HT (≤ 0.394). For the other three traits, prediction accuracies from highest to lowest were HW > HL > HN.

### Cross validation across subpopulations

**Figure 6:**
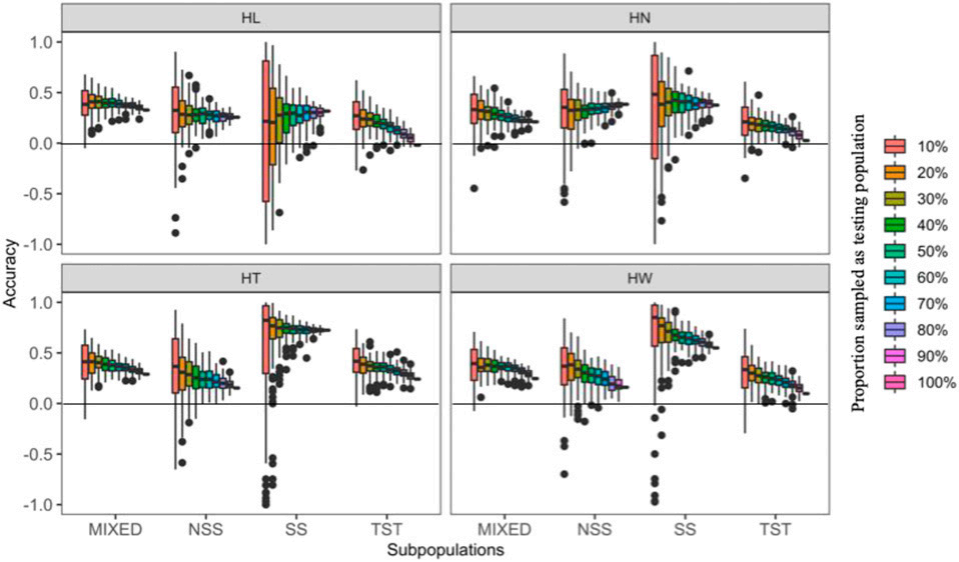
Prediction accuracies for husk traits across subpopulations. There are 67 inbred lines in non-stiff stalk (NSS), 27 in stiff stalk (SS), 193 in tropical-subtropical (TST) and 211 in admixed (MIXED). For each subpopulation, certain proportions (10–90%) of inbred lines were sampled as testing population and the rest, including the lines from other subpopulations, as the training population. The sampling was replicated 100 times. Husk traits: HL = husk length, HN = husk layer number, HT = husk thickness, and HW = husk width.

We also assessed prediction accuracy by masking varying proportions (10–100%) of the lines from each of the four subpopulations into the testing population and treating the remaining lines as the training population (Figure 6 and Table S3). For example, the 10% proportion of inference within MIXED subpopulation represents sampling 10% lines from this subpopulation as testing population and remaining 90% lines from MIXED subpopulation plus all the lines from other three subpopulations were treated as training population.

In the MIXED subpopulation, prediction accuracy varied little among husk traits. In order from highest to lowest, the prediction accuracies for the four husk traits were HL > HT > HW > HN. In the NSS subpopulation, from highest to lowest, prediction accuracies were HW > HN > HT > HL. In the SS subpopulation, HT exhibited the highest prediction accuracy (≥0.718); the other three traits ordered as HW > HN > HL. In the TST subpopulation, HT exhibited the highest prediction accuracy; the order of prediction accuracies for the other traits was HW > HL > HN. For HL, the highest prediction accuracy occurred in MIXED. For the other three husk traits, their highest prediction accuracies occurred in SS.

## Discussion

### Heritability plays the key role for GS

Heritability is variously dependent on the genetic architecture of a trait. For example, in plants, flowering traits that are controlled by several major QTL have high heritabilities and yield traits that are controlled by multiple small-effect genetic loci have low heritabilities(Dicenta *et al.* 1993; Crossa *et al.* 2010; Heffner *et al.* 2011). In turn, trait heritability affects GS prediction accuracy(Zhang *et al.* 2017b). For example, GS with traits of higher heritability always results in a higher prediction accuracy compared to traits of lower heritability(Jannink *et al.* 2010). In our study, HT had the highest heritability across both planting locations and among all husk traits. On the contrary, HL had the lowest heritability in Beijing and the second lowest in Sanya. For the other two husk traits, GS prediction accuracy was better for HN than HW.

### Benefit of using other subpopulations depends on training population size

Kinship among individuals is critical for GS. To have a high prediction accuracy, individuals in the testing population must have closely related individuals in the training population. Population structure can be the major factor affecting kinship. For example, in a previous study with maize and rice diversity panels, population structure explained 33% and 7.5% of the genomic variation, respectively(Guo *et al.* 2014). Individuals within subpopulations are more related than individuals among the subpopulations. For such reason, closely related individuals within the same subpopulation structure were split across training and testing populations(Ly *et al.* 2013; Spindel *et al.* 2015).

Our association panel was clustered into four subpopulations, MIXED, NSS, SS, and TST based on origins. Population study using 1,536 SNPs suggested that TST had the largest distance with the rest, especially SS, on the first principal component which explained 18.2% of total genetic variation. The second principal component (6.9% variance explained) separates SS from NSS with MIXED in the middle among other three with majority between TST and NSS (Yang *et al.* 2011). We conducted two sampling schemes in cross validation to evaluate the relationship between GS prediction accuracy and the relatedness between training and testing populations. One sampling scheme was to evaluate how one subpopulation was influenced by other subpopulations (Guo *et al.* 2014). The other was to evaluate how a subpopulation performed without using other subpopulations (Ly *et al.* 2013; Guo *et al.* 2014). In both schemes, the SS subpopulation showed the highest prediction accuracies across all husk traits, except HL.

For a subpopulation, prediction accuracies were higher when training populations were large and sampled with the same subpopulation than introducing extra lines from other subpopulations. For examples, HT in SS had prediction accuracy of 0.634 with 90% of lines as training population. The prediction accuracy dropped to 0.542 when all lines from other subpopulations joined to the training population; HT in NSS had prediction accuracy of 0.394 with 90% of lines as training population. The prediction accuracy dropped to 0.321 when all lines from other subpopulations joined to the training population.

However, when the numbers of lines in the training population were small, introducing lines from other subpopulations was beneficial. For examples, HT in SS had prediction accuracy of 0.358 with 20% of lines as training population. The prediction accuracy increased to 0.724 when all lines from other subpopulations joined to the training population; HT in NSS had prediction accuracy of 0.088 with 20% of lines as training population. The prediction accuracy increased to 0.202 when all lines from other subpopulations joined to the training population.

### Implication to breeding on husk traits

Determining the most suitable husk traits for breeding improvements in maize depends primarily on planting location, especially climatic conditions. For example, in temperate areas such as north China, appropriate husk traits would include a shorter length, a lower number of layers, a thinner cross-section (thickness), and a narrower width, all features conducive to fast kernel dehydration during mechanical harvest. According to GEBVs of husk traits (supplemental material Table S4 and S5) and the relationships between husk traits and PCs (Figure S2), TST lines are unfavorite due to high values on HT and HN for temperate areas. SS, NSS, and MIXED lines are in favorite.

In contrast, in tropical and subtropical areas, such as southern China, appropriate husk traits would include greater length, number of layers, thickness, and width. Together, these characteristics are suitable for protection against pest damage and pathogen infection, which are more prevalent and intense in tropical and subtropical areas(Renfro and Ullstrup 1976; Afolabi *et al.* 2007). Most lines in the TST subpopulation should be appropriate choices for breeding improvements in tropical and subtropical areas.

Trait wise, the most predictable husk trait in our GS study was HT. Consistent with its high heritability in all locations, HT exhibited the highest prediction accuracies within and among most subpopulations. Thus, to improve husk traits by GS in maize breeding selection programs, we recommend beginning with HT.

## Conclusions

The four husk traits had moderate to high heritabilities with HT at the top. The higher heritability, the higher accuracy of genomic prediction. Among four GS models, gBLUP with PCs has the highest prediction accuracies, followed by gBLUP without PCs, PCs only. With the best model of gBLUP with PCs, including individuals of external subpopulations, would help to predict individuals in a subpopulation when the training individual within the subpopulation was not large. Otherwise, the inclusion of the individuals of external subpopulation decreased prediction accuracy. HT was recommended as the first husk trait for breeding using GS.
